# Notch Blockade Specifically in Bone Marrow-Derived FSP-1-Positive Cells Ameliorates Renal Fibrosis

**DOI:** 10.3390/cells12020214

**Published:** 2023-01-04

**Authors:** Yongdong Wu, Ming Liang, Fengzhang Huang, Owen H. Cheng, Xiaoguang Xiao, Tae Hoon Lee, Luan Truong, Jizhong Cheng

**Affiliations:** 1 Department of Nephrology, The Second Affiliated Hospital, School of Medicine, South China University of Technology, Guangzhou 510000, China; 2 Selzman Institute for Kidney Health, Section of Nephrology, Department of Medicine, Baylor College of Medicine, Houston, TX 77030, USA; 3 Department of Pathology, Houston Methodist Hospital, Houston, TX 77030, USA

**Keywords:** fibroblast specific protein-1 (FSP-1), fibrosis, unilateral ureter obstruction (UUO), Notch signaling pathway, macrophage

## Abstract

Background: The infiltration of inflammatory cells during a kidney injury stimulates myofibroblast activation leading to kidney fibrosis. Fibroblast-specific protein 1 (FSP-1) positive cells have been reported as either myofibroblasts or monocytes during tissue fibrosis. The functions of FSP-1^+^ cells that are associated with the development of renal fibrosis and the signaling pathways that regulate FSP-1^+^ cell activation have not been well defined. Methods: In mice with unilateral ureteral obstruction (UUO), we characterized FSP-1^+^ cells and determined the role of the Notch signaling pathway in the activation of bone marrow-derived FSP-1^+^ cells during kidney fibrosis. Results: In kidneys from mice with UUO, the FSP-1^+^ cells accumulated significantly in the tubulointerstitial area. By using immunostaining and FSP-1 reporter mice, we found that FSP-1 was co-stained with inflammatory cell markers, but not myofibroblast markers. Results from mice with bone marrow transplantations showed that FSP-1^+^ cells in obstructed kidneys represent a bone marrow-derived population of inflammatory cells. In cultured FSP-1^+^ cells, the inhibition of Notch signaling suppressed the activation and cytokine secretion of FSP-1^+^ cells that were induced by LPS but not by IL-4. The specific KO or blockade of Notch signaling in bone marrow-derived FSP-1^+^ cells suppressed UUO-induced ECM deposition, the infiltration of FSP-1^+^ inflammatory cells, and cytokine production. These responses ameliorated myofibroblast accumulation and renal fibrosis in obstructed kidneys. Conclusion: Our study reveals that most FSP-1^+^ cells in obstructed kidneys are activated macrophages that are derived from bone marrow and that Notch signaling activates the production of M1 cytokines in FSP-1^+^ monocytes/macrophages, which is important for renal inflammation and fibrosis.

## 1. Introduction

Kidney fibrosis from chronic injuries is characterized by the presence of interstitial fibroblasts and a decline in renal function. Histological, inflammatory lesions usually involve recruitment, immune activation, and proliferation of leukocytes [1]. In addition, the accumulation of mononuclear inflammatory cells in the damaged renal interstitium is a universal finding in chronic kidney disease (CKD). Macrophages seem to foster all these morphological abnormalities, which suggests the apathogenic role of inflammation in the development of chronically scarred kidneys [2], but their specific contributions remain controversial.

Fibroblast-specific protein 1 (FSP-1), also known as S100A4, is expressed in fibroblasts in different organs that undergo tissue remodeling, including the kidneys, lungs, and heart [3,4,5]. In addition, FSP-1 is commonly used as a marker to identify epithelial cells undergoing epithelial-mesenchymal transition (EMT) during tissue fibrogenesis. However, there is evidence that challenges the specificity of FSP-1 as a fibroblast marker because the majority of FSP-1^+^ cells in the infarcted myocardium are identified as hematopoietic cells [6]. In other models of fibrosis occurring in human and experimental liver disease, FSP-1-positive cells express F4/80 and other markers of the myeloid lineage [7]. Although FSP-1 upregulation has been demonstrated in renal fibrosis [8], the majority of FSP-1^+^ cells express markers of mononuclear cells in the interstitium of injured kidneys [9]. Consequently, using FSP-1/S100A4 as a (myo)fibroblast marker has been challenged [10]. Moreover, the function of FSP-1^+^ cells in renal fibrosis has not been fully understood.

Notch signaling is a critical regulator of cell fate during kidney development [11]. It is also a central regulator of cellular function in pathological conditions. Activation of Notch signaling and increased transcription of Notch target genes are found in kidney injuries [12,13]. Treatment with γ-secretase inhibitor blocks Notch activation and prevents the development of kidney fibrosis after the kidney injury [14]. In contrast, overexpression of the Notch intracellular domain (NICD) in cultured renal cells could elicit different biological responses, including cell proliferation, apoptosis, inflammatory, and profibrotic responses. Notably, the inactivation of Notch 1 or Notch 2 facilitates cyst formation, while overexpression of N1ICD in podocytes or tubular cells promotes glomerulosclerosis and interstitial fibrosis, respectively [15,16].

Several studies also show that Notch signaling plays an important role in inflammatory disorders [17]. A role for Notch signaling is present during the recruitment and function of macrophages at sites of inflammation via a mechanism that induces CCR2 and expression of other cytokines. However, the role of FSP-1 cells in the development of renal fibrosis and whether Notch signaling promotes its activation have not been studied.

In this study, we showed the accumulation of FSP-1^+^ cells in obstructed kidneys and characterized FSP-1^+^ cells by using reporter mice and BM transplantations. The roles of the Notch signaling pathway in the activation and differentiation of FSP-1^+^ cells were explored in mice with a loss-of-Notch function.

## 2. Methods

### 2.1. Animals

All studies were approved by the Institutional Animal Care and Use Committee of Baylor College of Medicine, Houston, Texas, USA, and they were performed in accordance with the National Institutes of Health guidelines. Mice were housed in an animal facility with a 12 h light/dark cycle. FSP-1-Cre mice and FSP-1-GFP transgenic mice were purchased from Jackson Laboratories (Bar Harbor, ME, USA). RBP-Jκ^f/f^ mice were generously provided by Dr. Susztak from Albert Einstein College of Medicine, New York, USA and with permission from T. Honjo (Kyoto University Faculty of Medicine, Kyoto, Japan). To generate mice with KO of RBP-Jκ in FSP-1 cells, mice with a floxed RBP-Jκ allele were bred with FSP-1-Cre mice. After a backcross, RBP-Jκ^f/f^/FSP-1-Cre (FSP-1^RBP-Jκ KO^) mice were obtained (Supplemental Figure S1). Male and female FSP-1^RBP-Jκ KO^ mice and littermate control (FSP-1-Cre negative) mice were studied.

### 2.2. DNMAML1 Transgenic Mice

We used the TetOn/rtTA (reverse tetracycline transactivator) inducible system and Flox-Cre recombination techniques to create FSP-1 cell-specific, inducible DNMAML1 (Dominant Negative Form of Mastermind-like Protein 1, DNMAML1) transgenic mice. To breed these mice, three strains of mice were required (Supplemental Figure S2): (1) FSP-1-Cre mice (Jackson lab), that constitutively expressed Cre recombinase, driven by the tissue-specific promoter in FSP-1 cells; (2) rtTA-EGFP transgenic mice (Jackson lab), in which the expression of floxed-*rtTA* is under the control of the ubiquitously expressed *ROSA26* promoter; a floxed stop cassette present between the *ROSA26* promoter and *rtTA*, confined rtTA expression to the cells in which Cre recombinase was present; (3) the TetO-DNMAML1-GFP transgenic mice (Jackson lab), driven by the tetracycline-inducible promoter (*tetO*). The genotyping was performed according to the protocol provided by the Jackson Laboratory. The resulting mouse line hereafter was referred to as dnMAML1/FSP-1 mice (FSP-1^DNMAML1^ mice). Transgenic littermates were treated with doxycycline-containing water (0.5 mg/mL; Sigma Chemicals, with 5% sucrose added) to induce DNMAML1 expression. DNMAML-negative mice that were treated with doxycycline were used as control. The expression of DNMAML1 was determined by Western blots.

### 2.3. Generation of Reagents

Penicillin, streptomycin, tetracycline, Dulbecco’s Modified Eagle’s medium, and fetal bovine serum were obtained from Invitrogen (Life Technologies, Carlsbad, CA, USA). The protein assay kit was from Bio-Rad (Hercules, CA, USA). Fibronectin and S100A4 were from Sigma-Aldrich (St. Louis, MO, USA); CD31 antibodies were from BD Biosciences (BD Biosciences, San Jose, CA, USA); antibodies against Jagged 1, and Hes 1, Hey 1, and Hes 5 were obtained from Santa Cruz Biotechnology (Santa Cruz, CA, USA). Antibodies against Notch 1, N1ICD, RBP-Jκ, and iNOS were from Cell Signaling Technology (Danvers, MA, USA). The fluorescent-700/800 secondary antibodies were obtained from Invitrogen (Carlsbad, CA, USA), an antibody against CD45 was from Millipore (Billerica, MA, USA), and antibodies against F4/80, proliferating cell nuclear antigen, GFP, and rabbit anti-α-SMA were from Abcam (Cambridge, MA, USA). The GFP antibody was purchased from Rockland Immunochemicals (Limerick, PA, USA), and lipopolysaccharide and recombinant IL-4 were purchased from R&D Systems (Minneapolis, MN, USA). BrdU Labeling and Detection Kits were obtained from Roche (Indianapolis, IN, USA).

### 2.4. Renal Interstitial Fibrosis Model

UUO or sham surgeries were performed on two-to-three-month-old mice, as described previously [18,19]. Mice (6 in each group) were anesthetized with an intraperitoneal injection of Rodent III Combo. Kidney tissues were harvested 3 or 7 days after UUO or sham surgery. Because previous studies showed no differences between these time points in sham-operated mice [19], a single time point (day 3 or 7) was used for sham controls in each experiment. Under anesthesia, the left ureter was isolated and ligated for 3 or 7 days. After 3 or 7 days, all animals were euthanized, and the kidneys were analyzed.

### 2.5. Histology and Immunohistochemistry

For histological analysis, the kidneys were prepared by perfusion of the mice with PBS through the left ventricle, and slides of the kidneys were prepared as described.

Double immunofluorescence staining was performed as a previous report. Isotype-matched IgG or PBST was used as the negative control. The areas of positive signals were measured using the NIS-Elements BR 3.0 program. Picrosirius Red and Trichrome staining were performed for assessment of collagen deposition as described. The amount of cortical fibrillary collagen was determined by observing areas stained with picrosirius red with a Nikon Eclipse 80i fluorescence micro microscope (Melville, NY, USA). Images from each section were analyzed in a blind manner and quantified using Image-Pro Plus software (Media Cybernetics, Silver Spring, MD, USA).

### 2.6. Isolation of BM–Derived FSP-1^+^ and BM Transplantation

BM cells were obtained from mouse tibias and femurs of FSP-1–GFP transgenic mice. GFP^+^ BM cells were isolated by cell sorting (Becton-Dickinson LSRII flow cytometer, BD Biosciences, San Jose, CA, USA). BM transplantation was performed by injecting 5 × 10^6^ BM cells into the lateral tail vein of lethally irradiated (1100 rads) mouse recipients [20].

### 2.7. Isolation of Renal Cells from Control and Obstructive Kidneys

UUO (n = 6 mice) and sham (n = 6 mice) surgeries were performed, and renal single cells were isolated and analyzed by flow cytometry. Briefly, renal tissue was minced and placed into a cocktail of 0.25 mg/mL Liberase Blendzyme 3 (Roche Applied Science), 20 U/mL DNase I (Sigma-Aldrich) and shaken at 37 °C for 20 min. Subsequently, cells were passed through 40 μm nylon mesh and centrifuged (10 min, 200 g, 4 °C). Cells were stained and analyzed using flow cytometry. The following dyes and antibodies were used: APC-Cy7-conjugated anti-CD45, Alexa Fluor 700-conjugated anti-CD3 (both from BD Pharmingen), PerCP/Cy5.5-labeled anti-CD31, PE/Cy7-labeled anti-F4/80 (both from Biolegend), and Cy3-conjugated anti-α-SMA (Sigma). Stained single cells were resuspended in a staining buffer and immediately analyzed with a Becton Dickinson LSRII flow cytometer (BD Biosciences).

### 2.8. BrdU Experiments

BrdU was administered to the mice (i.p., 1.5 mg/day) for 3 or 7 days before kidney collection. BrdU immunofluorescent staining was performed according to the manufacturer’s instructions (BrdU Labeling and Detection Kit, Roche, Indianapolis, IN, USA).

### 2.9. Cell Culture

To evaluate cytokines production in GFP^+^ BM cells, LPS (100 ng/mL) or IL-4 (40 ng/mL) were added and cultured for 72 h before the cells were harvested for further analysis.

### 2.10. Measurements of mRNA Expression

Total RNAs were isolated by RNeasy kit (Qiagen, Valencia, CA, USA). Real-time reverse transcriptase–polymerase chain reactions were performed with primers (Supplemental Table S1).

### 2.11. Western Blot Analysis

The protein content of cell extracts prepared in radioimmunoprecipitation assay buffer was determined by using the Bradford protein assay kit (BioRad, Hercules, CA, USA). About 30 mg of heat-denatured proteins were separated by sodium dodecyl sulfate-polyacrylamide gel electrophoresis and blotted onto nitrocellulose membranes. Subsequently, the immunoblots were blocked with 5% skimmed milk in a Tris-buffered saline solution, and immunoblots were probed separately with various primary antibodies. Fluorescently labeled secondary antibodies were detected by the Odyssey Infrared Imaging System (LI-COR Biosciences, Lincoln, NE, USA).

### 2.12. Statistical Analysis

All statistical analyses were performed with at least 3 independent biological or experimental replicates. Statistical analyses were performed in Graphpad Prism Version 8. Scatter dot-plots and error bars represent the mean ± SD. Significant differences were determined by Mann–Whitney test and unpaired Student’s *t*-test when comparing two groups; one-way or two-way ANOVA was used to compare multiple groups. Statistical tests were described in each figure legend. Differences were considered statistically significant at *p*-value < 0.05.

## 3. Result

### 3.1. FSP-1 Positive Cells Accumulate in Obstructed Kidneys

**Figure 1:**
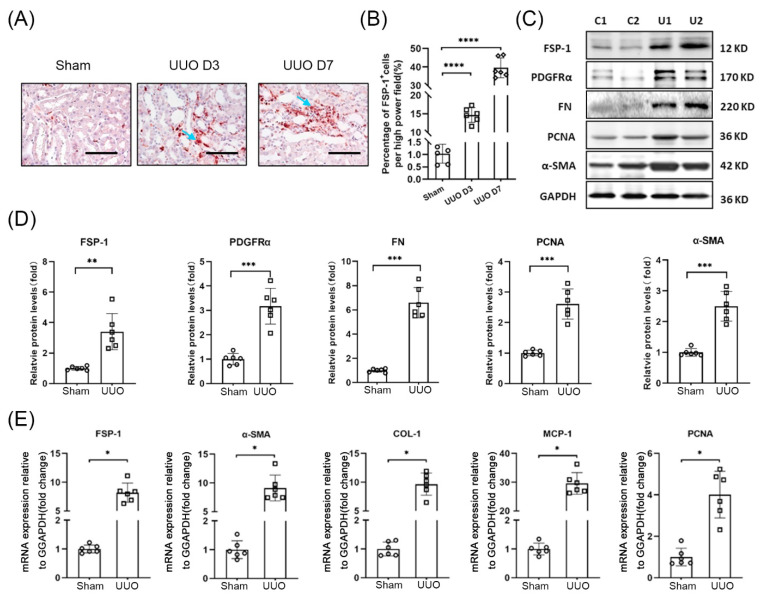
**FSP-1 positive cells accumulate in UUO.** (**A**,**B**). The presence of FSP-1^+^ cells was determined by immunostaining in kidneys after UUO on day 3 and day 7. Positive cells (FSP-1^+^) were indicated by arrows (**A**) and quantified (**B**) (****, *p* < 0.0001 vs. Sham; bar = 50 μm). (**C**,**D**). The expression and densitometric analysis of the indicated markers in control and obstructed kidneys (**, *p* < 0.005; ***, *p* < 0.001). (**E**). Real-time RT-PCR analysis of the expression of indicated molecules. Relative mRNA levels were shown after normalization with GAPDH. (*, *p* < 0.05). C, sham controls; U, UUO kidney; GAPDH, glyceraldehyde-3-phosphate dehydrogenase; PDGFR, platelet-derived growth factor receptor; SMA, smooth muscle actin; FN, fibronectin; PCNA, proliferative cell nuclear antigen. Data are expressed as mean ± SEM, n = 6 mice.

In UUO mice, FSP-1 expression was increased significantly on day three after UUO and was even more pronounced after day seven (Figure 1A). In healthy mouse kidneys, only a few FSP-1^+^ cells were found. While many FSP-1^+^ cells were found in the tubulointerstitial area in obstructed kidneys (arrowheads pointed in Figure 1A), the number of FSP-1^+^ cells increased over time in the UUO models, which were evaluated by immunohistochemistry (Figure 1B).

The expression of fibroblast markers (FN, PDGFRα, α-SMA) and cell proliferation marker PCNA was also increased significantly in kidneys on day seven of mice with UUO (Figure 1C,D). The mRNA levels of FSP-1, α-SMA, Collagen I, MCP-1, and PCNA were significantly increased in the obstructed kidneys vs. in the kidneys of sham control mice (Figure 1E).

### 3.2. FSP-1 Is Not a Specific Marker for Myofibroblasts in Fibrogenic Kidneys

**Figure 2:**
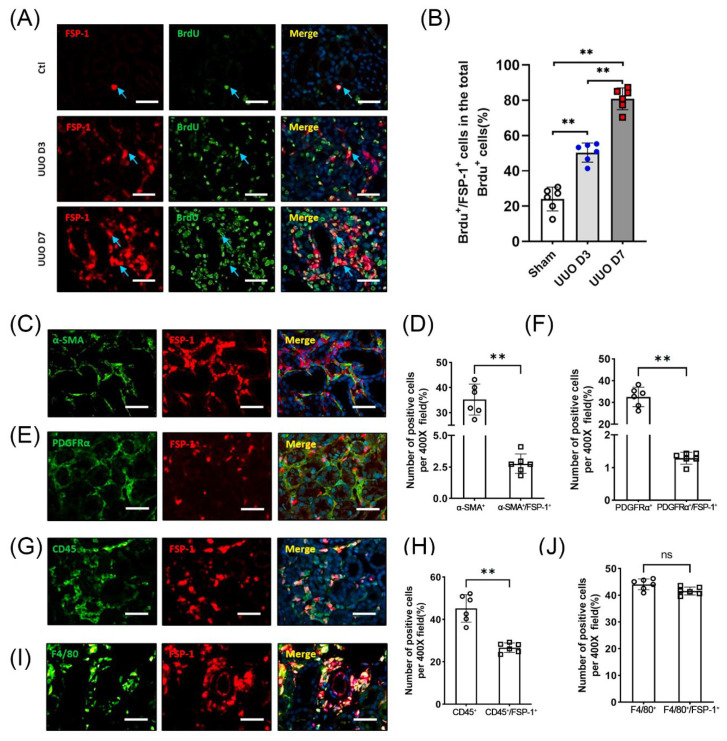
**FSP-1 is not a specific marker for myofibroblasts.** (**A**,**B**), Double immunostaining of FSP-1 (red) and BrdU (green) was performed to detect proliferation of FSP-1^+^ cells in WT mice following UUO on day 3 and 7. Double positive cells (FSP-1^+^/BrdU^+^) were indicated by arrows (blue) and quantified in Panel (**B**). (**C**,**D**). Double immunostaining of FSP-1 (red) and α-SMA (green) in the obstructed kidneys. The percentage of the number of all α-SMA^+^ cells and α-SMA^+^/FSP-1^+^ cells in the obstructed kidneys was shown. (**E**,**F**). Double immunostaining of FSP-1 (red) and PDGFRα (green) in the obstructed kidneys. The percentage of the number of all PDGFRα^+^ cells and PDGFRα^+^/FSP-1^+^ cells in the obstructed kidneys was shown. (**G**,**H**). Double immunostaining of FSP-1 (red) and CD45 (green) in the obstructed kidneys. The percentage of the number of all CD45^+^ cells in the CD45^+^/FSP-1^+^ cells was shown. (**I**,**J**). Double immunostaining of FSP-1 (red) and F4/80 (green) in the obstructed kidneys. The percentage of the number of all F4/80^+^ cells and F4/80^+^/FSP-1^+^ cells in the obstructed kidneys was shown. BrdU, 5-bromodeoxyuridine; PDGFR, platelet-derived growth factor receptor; SMA, smooth muscle actin. Bar = 50 μm in panels (**A**,**C**,**E**,**G**,**I**); data are expressed as mean ± SEM, n = 6 mice. (**, *p* < 0.005; ns, no significance).

To determine the renal cell proliferation responses after UUO injury, BrdU was injected into mice to label proliferating cells. Immunostaining results showed that BrdU^+^/FSP-1^+^ cells were most pronounced in the interstitium (Figure 2A). BrdU^+^ cells were also detected in the dilated tubular cells. Quantification results showed that > 95% of interstitial FSP-1^+^ cells were positive for BrdU staining in the kidneys of WT mice on the seventh day following UUO (Figure 2B).

We examined whether FSP-1 serves as a marker for myofibroblasts. The double immunofluorescence staining of FSP-1 with mesenchymal markers (including PDGFRα) and α-SMA was performed. The results showed that the cell staining positively for PDGFRα or α-SMA represented only 1.5% or 4.1% of FSP-1 positive cells, respectively (Figure 2C–F). In contrast, almost all FSP-1 positive cells in obstructed kidneys were positively stained with the hematopoietic cell marker CD45^+^ (Figure 2G,H). Notably, a significant proportion of FSP-1 positive cells were found to express F4/80^+^, a macrophage marker (Figure 2I,J). These results indicate that FSP-1^+^ cells in obstructed kidneys express markers of hematopoietic cells, but not markers of fibroblasts.

### 3.3. The Majority of FSP-1^+^ Cells in the Obstructed Kidneys Are Identified as BM-Derived Hematopoietic Cells

**Figure 3:**
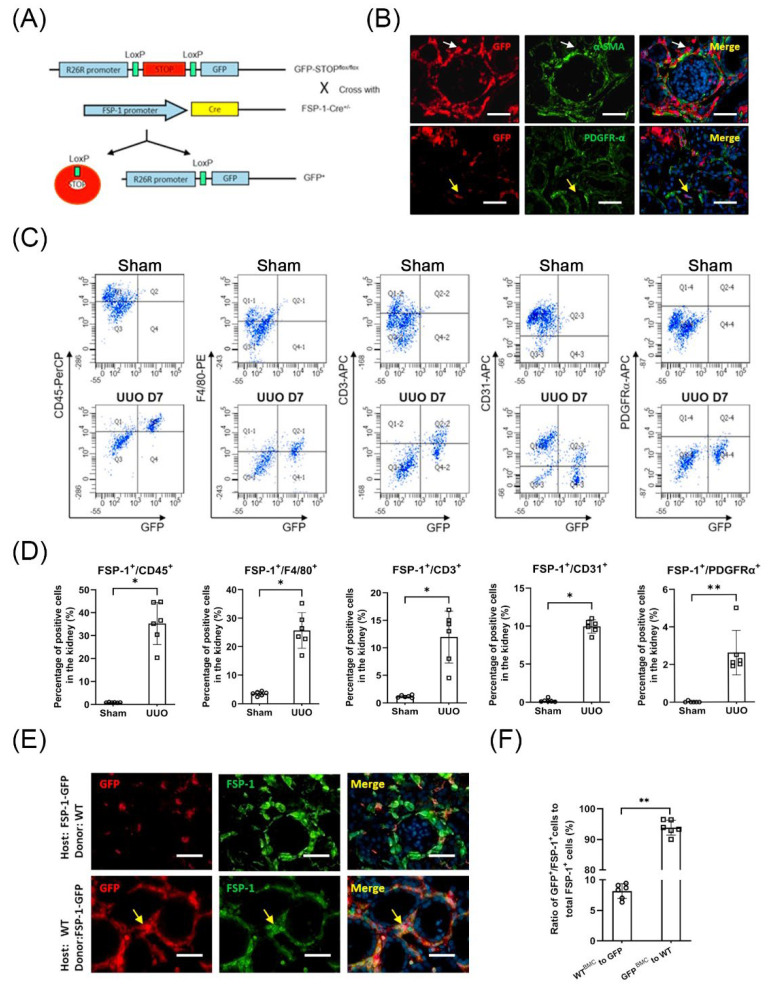
**Characterization of FSP-1^+^ cells in obstructed kidneys.** (**A**). Schematic of breeding of FSP-1^GFP^ reporter mice. (**B**). Double immunostaining of GFP with α-SMA or PDGFRα in obstructed kidneys of FSP-1^GFP^ mice. The arrows indicate that the GFP^+^ cells were not co-localized with α-SMA^+^ or PDGFRα^+^ cells. (**C**,**D**). Flow cytometry and quantification analysis of FSP-1^+^ cells. Most FSP-1^+^ cells were positive for CD45^+^ (hematopoietic cell marker) or F4/80^+^ (macrophage marker) in obstructed kidneys after 7 days of UUO. (*, *p* < 0.05 vs. sham; **, *p* < 0.005). (**E**,**F**). FSP-1^+^ cells found in UUO are BM-derived. Double Immunostaining of GFP and FSP-1 was performed in the obstructed kidneys of WT mice transplanted with BM from an FSP-1^GFP^ mouse or FSP-1^GFP^ mouse transplanted with BM from WT mice (yellow arrows point to FSP-1^+^/GFP^+^ double-positive cells (**E**). The ratio of GFP^+^/FSP-1^+^ double-positive cells in obstructed kidneys was counted and calculated (**F**) (**, *p* < 0.005). Bar = 50 μm in panels (**B**,**E**); data are expressed as mean ± SEM, n = 6 mice; GFP, green fluorescent protein; PDGFR, platelet-derived growth factor receptor; SMA, smooth muscle actin.

To track the FSP-1 cells in injured kidneys, FSP-1-GFP reporter mice, in which the FSP-1 promoter drives GFP expression, were subjected to UUO (Figure 3A). Cells producing GFP positive signals represent FSP-1 expressing cells in FSP-1-GFP reporter mice. Cells that were positive for α-SMA or PDGFRα in obstructed kidneys amounted to only a small percentage of GFP-positive cells (Figure 3B). Flow cytometry results showed that FSP-1^+^ cells were significantly increased in kidneys after seven days following UUO but were absent in sham control mice. Nearly 95% of GFP-positive cells were CD45-positive (Figure 3C,D). Most FSP-1^+^ cells were positive for F4/80^+^ (macrophage marker) or CD45^+^ (hematopoietic cell marker) in obstructed kidneys after seven days of UUO. While only 2.15% of FSP-1^+^ cells expressed the myofibroblast marker PDGFRα^+^ (Figure 3C,D). These results indicate that the majority of FSP-1^+^ cells in UUO were F4/80^+^ macrophages.

To assess the contribution of BM-derived FSP-1^+^ cells to the total FSP-1^+^ population in obstructed kidneys, we performed BM transplantation. WT mice were transplanted with BM cells from FSP-1-GFP mice, and FSP-1-GFP mice were transplanted with WT BM cells. Immunostaining for FSP-1^+^ cells (green) and GFP^+^ cells (red) indicated that >90% of the FSP-1^+^ cells were BM-derived in the fibrotic kidneys, whereas <10% of the FSP-1^+^ cells were derived from the resident cells (Figure 3E,F).

### 3.4. Notch Signaling Is Activated during UUO

**Figure 4:**
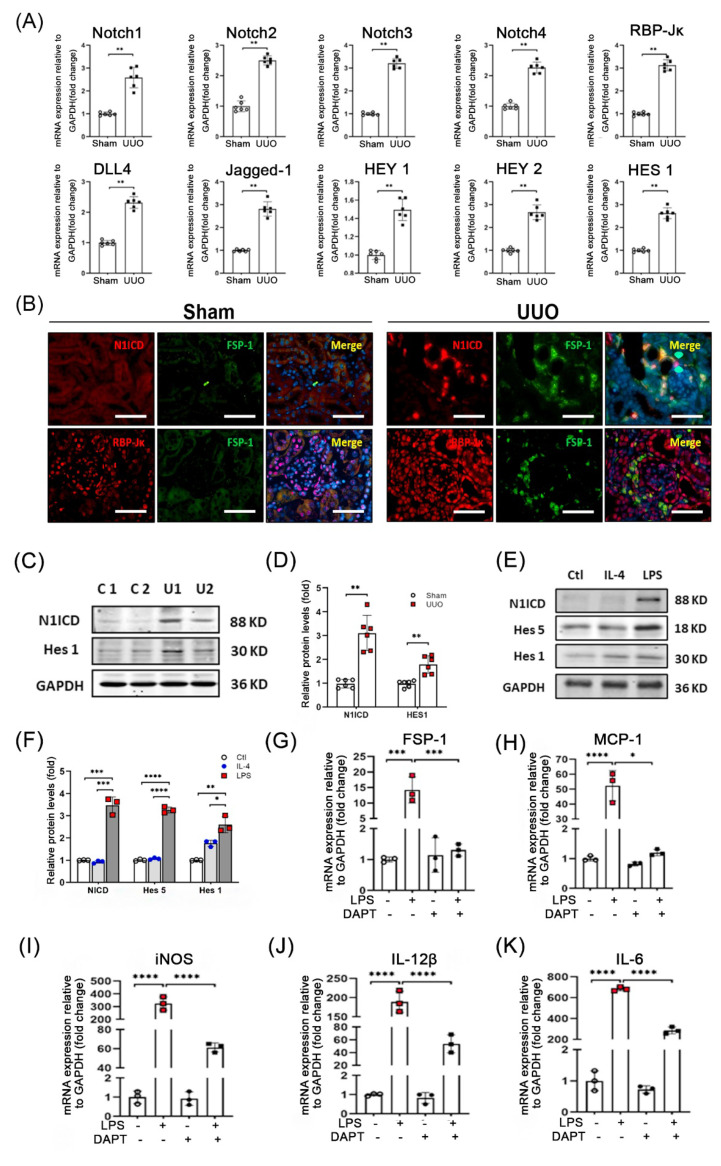
**Notch signaling pathway was activated in kidneys after UUO.** (**A**). Relative mRNA levels of molecules in Notch signaling pathway were determined by qRT-PCR in mice 7 days following UUO. Gene expression levels were normalized to mRNA levels of control animals, and significance was calculated (**, *p* < 0.005). (**B**). Double immunostaining of FSP-1 with N1ICD or RBP-Jκ was performed in sham control and obstructed kidneys (bar = 50 μm). (**C**,**D**). Expression of N1ICD and Hes1 were determined by Western blotting and density analysis in obstructed kidneys on day 7 (**, *p* < 0.005 vs. Ctl). (**E**,**F**). Western blotting and density analysis of Notch signaling in FSP-1^+^ BM cells from FSP-1^GFP^ mice after stimulation with IL-4 or LPS. (*, *p* < 0.05; **, *p* < 0.005; ***, *p* < 0.001; ****, *p* < 0.0001; vs. LPS-treated group; n = 3 repeats). (**G**–**K**). The expression of cytokines of type 1 macrophages was determined by qRT-PCR after the FSP-1 cells were treated with LPS and DAPT. (*, *p* < 0.05; ***, *p* < 0.001; and ****, *p* < 0.0001 vs. Ctl). Data are expressed as mean ± SEM, n = 6 mice in panels (**A**–**E**). Ctl, control; GFP, green fluorescent protein; N1ICD, NOTCH 1 Intracellular Domain; LPS, Lipopolysaccharide; DAPT, N-[N-(3, 5-difluorophenacetyl)-l-alanyl]-s-phenylglycinet-butyl ester.

Since the Notch pathway controls cell self-renewal, proliferation, and differentiation [15,16], we determined if Notch signaling regulates FSP-1 cell differentiation. Results from real-time RT-PCR analysis showed that the expression of Notch receptors and Notch ligands (Jag1 and Dll4) were induced in obstructed kidneys. Moreover, Notch target genes (Hes 1, Hey 1, and Hey 2) and the Notch transcription factor, RBP-Jκ, were also up-regulated in UUO (Figure 4A).

Immunostaining results showed that activated Notch 1 intracellular domain (N1ICD) was detected in dilated tubules and tubulointerstitial cells in UUO (7 day) mice, but there was no detectable N1ICD in the control kidneys (Figure 4B). More staining of activated Notch 1 and RBP-Jκ were detected in the nuclei of FSP-1^+^ cells in obstructed kidneys compared with sham control kidneys (Figure 4B). Consistently, protein levels of NI1CD and Hes1 were up-regulated in UUO mice suggesting that Notch/RBP-Jκ signaling is activated in FSP-1^+^ cells following UUO injury (Figure 4C,D).

To investigate the involvement of Notch signaling in the regulation of the differentiation of FSP-1^+^ BMs, we isolated the FSP-1^+^ cells from the BM of FSP-1-GFP mice by cell sorting. LPS or IL-4 treatment were used to induce the differentiation of BM cells into the type 1 macrophage (M1) or type 2 macrophage (M2), respectively. After overnight incubation, we found that the LPS-mediated differentiation of BM cells into M1 cells was associated with the upregulation of N1ICD, Hes5 and Hes1 (Figure 4E,F). LPS-treated BM cells showed increased mRNA levels of FSP-1 and inflammatory cytokines (MCP-1, iNOS, IL-12β and IL-6). The induction of these cytokines was diminished by the treatment of the Notch signaling inhibitor, DAPT (Figure 4G–K). These results demonstrate that Notch signaling is required for the activation and differentiation of BM-derived FSP-1 cells into M1 macrophages.

### 3.5. Genetic Ablation of RBP-Jκ in FSP-1^+^ Cells Suppresses Tubulointerstitial Fibrosis Caused by UUO

During canonical Notch signaling, the N1ICD translocates to the nucleus and binds to the transcriptional repressor, RBP-Jκ, converting it into an activator and inducing the expression of downstream target genes. RBP-Jκ is the only transcriptional coactivator that transactivates the signals of all four Notch receptors. To determine the role of Notch signaling in activating FSP-1^+^ macrophages, we generated conditional RBP-Jκ KO mice (RBP-Jκ^f/f^/FSP-1-Cre^+^), where the RBP-Jκ was KO only in FSP-1^+^ cells (Supplemental Figure S1A).

**Figure 5:**
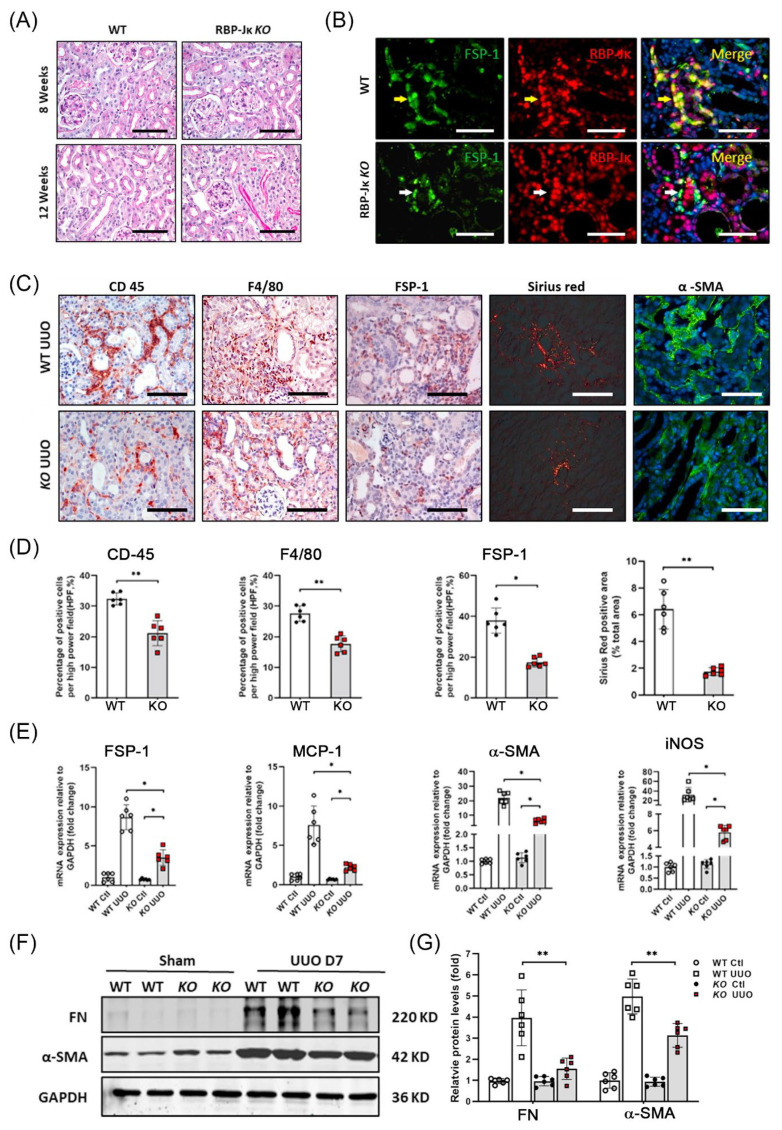
**Loss of RBP-Jκ-dependent Notch signaling in FSP-1^+^ cells ameliorates UUO-induced tubulointerstitial fibrosis.** (**A**). PAS staining of kidneys from WT and FSP-1^RBP-Jκ KO^ control mice at the age of 8 weeks and 12 weeks. (**B**). Representative picture showing the double immunofluorescent staining of FSP-1 and RBP-Jκ in kidneys of WT and FSP-1^RBP-Jκ KO^ mice at 1 week after UUO. Yellow arrows point to RBP-Jκ^+^/FSP-1^+^ inflammatory cells in the kidneys of WT mice, and white arrows point to RBP-Jκ^−^/FSP-1^+^ cells in the kidneys of FSP-1^RBP-Jκ KO^ mice. (**C**,**D**). Representative staining and density analysis of PAS, Sirius red, and the indicated markers in both WT and FSP-1^RBP-Jκ KO^ mice after UUO (*, *p* < 0.05, **, *p* < 0.01 vs. WT). (**E**). Real-time RT-PCR analysis of expression of indicated molecules (*, *p* < 0.05). (**F**,**G**). Representative western blotting and relative intensity analysis indicated levels of myofibroblast markers and Hes1 in obstructed kidneys of WT and FSP-1^RBP-Jκ KO^ mice (**, *p* < 0.05). GAPDH, glyceraldehyde-3-phosphate dehydrogenase; FN, fibronectin; SMA, smooth muscle actin; WT, wild type; KO, knock out. Bar = 50 μm in panels (**A**–**C**); data are expressed as mean ± SEM, n = 6 mice.

Results from real-time PCR and western blot analysis showed that RBP-Jκ was deleted in most BM-derived FSP-1 cells in FSP-1^RBP-Jκ KO/GFP^ mice (Supplemental Figure S1B,C). There was no apparent abnormality in the kidneys between WT and FSP-1^RBP-Jκ KO^ mice (Figure 5A). Results of double immunofluorescent staining showed that RBP-Jκ was not detected in FSP-1^+^ cells in the obstructed kidneys of FSP-1^RBP-Jκ KO^ mice (white arrow pointed, Figure 5B). There was an increase in the infiltration of leukocytes, deposition of interstitial collagen, as well as the staining of Sirius red in WT UUO mice, while these pathological phenotypes were suppressed in obstructed kidneys in FSP-1^RBP-Jκ KO^ mice (Figure 5C). The influx of FSP-1-positive CD45^+^ and F4/80^+^ macrophages was decreased in mice with UUO on day seven in FSP-1^RBP-Jκ KO^ mice vs. WT mice (Figure 5C,D). The α-SMA positive area was less in obstructed kidneys in FSP-1^RBP-Jκ KO^ mice compared to that in WT mice, indicating KO of RBP-Jκ in FSP-1 cells decreased the accumulation of interstitial myofibroblasts in obstructed kidneys (Figure 5C).

The expressions of FSP-1, α-SMA, and proinflammatory cytokine MCP-1 and iNOS were decreased significantly in the obstructed kidneys of FSP-1^RBP-Jκ KO^ mice compared to the results of the WT mice (Figure 5E). Moreover, the results of the Western blotting showed that the specific KO of *RBP-Jk* in FSP-1 positive cells significantly decreased the protein levels of α-SMA and fibronectin in the obstructed kidneys vs. those in WT mice at seven days after UUO (Figure 5F,G). Together, these results indicate that KO of RBP-Jκ in FSP-1 cells suppresses proinflammatory responses and renal fibrosis induced by UUO.

### 3.6. BM-Derived FSP-1^+^ Cells Are Functionally Important for Renal Fibrosis

Inflammation is associated with the development of renal fibrosis; to assess the functional importance of FSP-1^+^ cells in fibrosis, we determine whether KO of RBP-Jκ in BM-derived FSP-1^+^ cells results in reduced renal inflammation and fibrosis. WT mice were transplanted with either BM of FSP-1^RBP-Jκ KO^ mice (WT^RBP-Jκ_KO BM^) or WT BM cells (WT^WT BM^). Similarly, FSP-1^RBP-Jκ_KO^ mice were transplanted with either BM of FSP-1^RBP-Jκ KO^ mice (KO^RBP-Jκ_KO BM^) or WT BM cells (KO^WT BM^).

**Figure 6:**
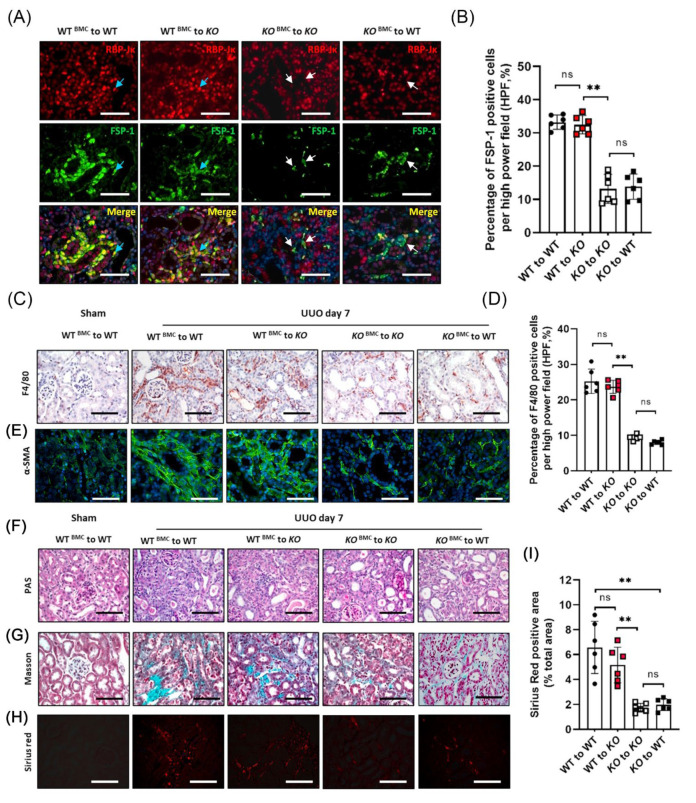
**RBP-Jκ KO in BM FSP-1^+^ cells suppresses UUO-induced inflammation and fibrosis.** (**A**,**B**). Double immunofluorescent staining of FSP-1 and RBP-Jκ in kidneys of BM transplanted chimeric mice after 7 days of UUO. Blue arrows point to FSP-1^+^/RBP-Jκ^+^ co-staining cells; white arrows point to FSP-1^+^/RBP-Jκ^−^ cells. Quantification of FSP-1^+^ cells of the immunostaining was shown in Panel B (**, *p* < 0.005). (**C**–**E**). Representative images of immunostaining of F4/80 and α-SMA in kidneys of BM transplanted chimeric mice after 7 days of UUO. Quantification of the immunostaining of F4/80^+^ cells was shown in Panel (**D**) (**, *p* < 0.005). (**F**,**G**). Histology (PAS staining) and Gomori’s Trichrome staining of kidneys of FSP-1^RBP-Jκ KO BM^ chimeric mice after 7 days of UUO. Controlled kidneys were from WT^WT BM^ mice. (**H**,**I**). Enhanced collagen deposition was determined by Sirius red staining (**, *p* < 0.005). The relative density analysis of Sirius red staining was shown in panel I. Bar = 50 μm in panels (**A**,**C**,**E**–**H**); data are expressed as mean ± SEM, n = 6 mice. ns: no significance.

The expression of RBP-Jκ in FSP-1^+^ cells was determined by double immunostaining; the results showed that RBP-Jκ was efficiently and specifically KO in obstructed kidneys in either WT^RBP-Jκ_KO BM^ mice or KO^RBP-Jκ_KO^ mice (Figure 6A). In contrast, the expression of RBP-Jκ was positively co-stained with FSP-1^+^ cells in WT^WT BM^ or KO^WT BM^ mice (Figure 6A). Notably, there was a significant reduction of FSP-1^+^ and RBP-Jκ^+^ cells in kidneys from WT^RBP-Jκ_KO BM^ mice and KO^RBP-Jκ_KO BM^ mice (Figure 6A,B). Moreover, the obstructed kidneys in WT^RBP-Jκ_KO BM^ mice showed less infiltration of F4/80 macrophages compared to that in WT^WT BM^ mice (Figure 6C,D), indicating that the recruitment of FSP-1^+^ cells from BM requires Notch signaling.

There were decreased A-SMA positive signals in obstructed kidneys in mice with BM from FSP-1^RBP-Jκ KO^ mice compared to the obstructed kidneys from mice with BM from WT mice (Figure 6E), indicating that accumulation of myofibroblast in obstructed kidneys is associated with active Notch signaling in BM-derived FSP-1 cells. The positive signals of PAS, Gomori’s Masson trichrome staining, and Sirius red in the kidneys were decreased on day seven of UUO in WT^RBP-Jκ_KO BM^ mice when compared to the kidneys from WT^WT BM^ mice (Figure 6F–I). Moreover, the levels of the Sirius red positive area which represents collagen deposition in KO^WT BM^ mice were similar to those in WT^WT BM^ UUO mice (Figure 6H,I), demonstrating that specific ablation of RBP-Jκ in BM-derived FSP-1^+^ cells reduced UUO-induced fibrosis.

Together, these results suggest that the recruitment of BM-derived FSP-1^+^ cells promotes kidney fibrosis, and RBP-Jκ deficiency in FSP-1^+^ BM cells suppresses the accumulation of BM-derived FSP-1^+^ cells in obstructed kidneys.

### 3.7. Overexpression of DNMAML1 in FSP-1^+^ Cells Inhibits UUO-Induced Renal Inflammation and Fibrosis

**Figure 7:**
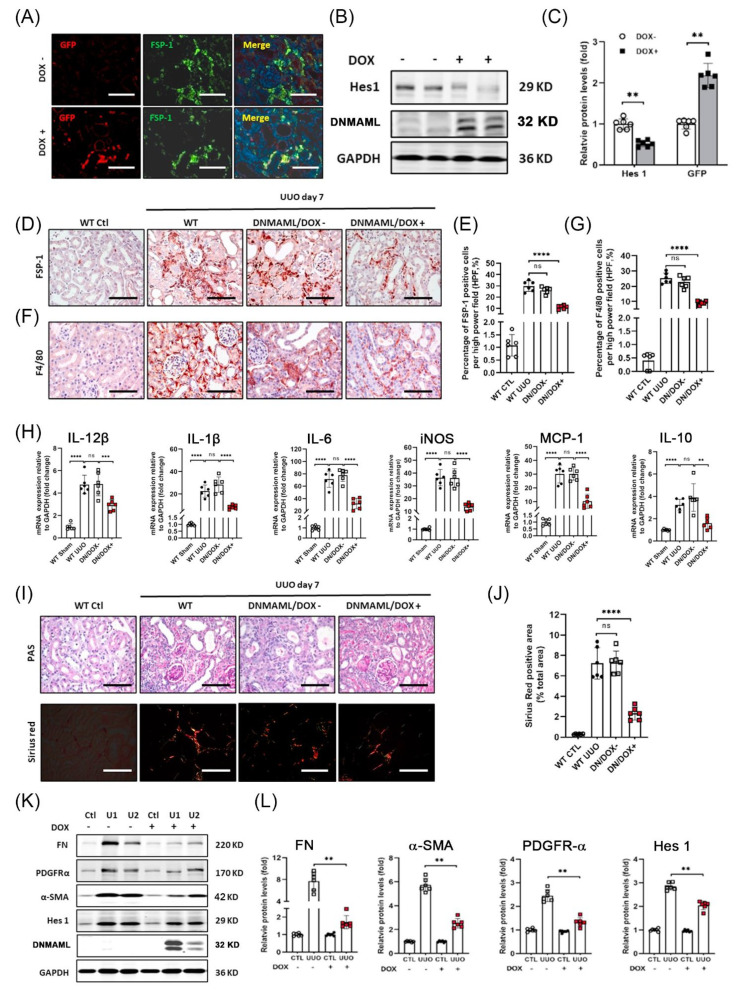
**DNMAML overexpression-mediated specific Notch blockade in FSP-1^+^ cells inhibits renal inflammation and fibrosis.** (**A**). Double fluorescent immunostaining of FSP-1/GFP in obstructed kidneys of mice with or without Dox induction. (**B**,**C**). Western blot and density analysis of expression of dnMAML1 and Hes1 in kidneys from FSP-1^DNMAML1^ mice with or without Dox induction at 7 days after UUO. (**, *p* < 0.05). (**D**–**G**). Immunostaining and densitometry quantification of expression of FSP-1 and F4/80 in kidneys of FSP-1^DNMAML1^ mice at 7 days after UUO (****, *p* < 0.0001). (**H**). qRT-PCR analysis of the mRNA levels of cytokines in WT and FSP-1^DNMAML1^ mice at 7 days after UUO (***, *p* < 0.001; ****, *p* < 0.0001). (**I**,**J**). PAS and Sirius red staining of kidneys of FSP-1^DNMAML1^ mice with or without Dox induction after 7 days of UUO. The relative density analysis of Sirius red staining was shown in Panel (**J**) (****, *p* < 0.0001). (**K**,**L**). The expressions of indicated molecules were detected in sham control and obstructed kidneys of FSP-1^DNMAML1^ mice by Western blot and density analysis. (mean ± SEM; **, *p* < 0.005). DN, Dominant Negative; DOX, Doxycycline; Ctl, control; GAPDH, glyceraldehyde-3-phosphate dehydrogenase; PDGFR, platelet-derived growth factor receptor; SMA, smooth muscle actin; FN, fibronectin; U, UUO sample. Bar = 50 μm in panels (**A**,**C**,**E**,**H**,**I**); data are expressed as mean ± SEM, n = 6 mice. ns: no significance.

Next, we used a tetracycline (Tet)-based inducible system to express a dominant-negative MAML1 fused to GFP (DNMAML1-GFP) to inhibit Notch signaling in FSP-1^+^ cells in mice with UUO (Supplemental Figure S2). Both FSP-1 promoter-driven Cre and the presence of doxycycline are required to induce DNMAML1-specific expression in FSP-1^+^ cells in FSP-1^DNMAML1^ mice. The results from immunohistochemical staining showed increased expression of DNMAML1/GFP in the FSP-1^+^ cells in obstructed kidneys of FSP-1^DNMAML1^ mice after doxycycline treatment (Figure 7A). The DNMAML1-GFP protein was only induced in obstructed kidneys in FSP-1^DNMAML1^ mice following doxycycline treatment (Figure 7B,C).

There was a significant reduction in inflammatory cell infiltration (F4/80^+^ macrophage and FSP-1^+^) in FSP-1^DNMAML1^ mice vs. results from non-dox-induced mice after UUO (Figure 7D–G). Similarly, the UUO-induced cytokine production was also significantly suppressed in FSP-1^DNMAML1^ mice (Figure 7H). The results from PAS and Sirius red staining confirmed that DNMAML1 overexpression in kidneys inhibited fibrosis on day seven of UUO when compared with control mice (Figure 7I,J). Consistent with these observations, reduced expressions of FN, α-SMA, and PDGFRα were found in FSP-1^DNMAML1^ mice (Figure 7K,L). As expected, the expression of Notch target gene Hes 1 was reduced in the kidneys of FSP-1^DNMAML1^ mice (Figure 7K,L). These results suggest that the upregulation of DNMAML1 expression in FSP-1^+^ cells suppresses inflammation and fibrosis after UUO.

## 4. Discussion

Although Notch function in kidney diseases has been reported [21,22], the mechanistic roles of Notch in regulating the activation and differentiation of monocytes/macrophages in renal inflammation and fibrosis is unclear. Our results demonstrate that BM-derived FSP-1^+^ cells stimulate the expression of proinflammatory cytokines in a fashion related to Notch/RBP-Jκ signaling. The following results support our conclusion: (i) results from BM transplantation experiments verify that BM-derived FSP-1^+^ cells are involved in the process of developing renal inflammation and fibrosis; (ii) specifically blockades of Notch/RBP-Jκ signaling in FSP-1^+^ cells inhibit macrophage infiltration and renal fibrosis in UUO; (iii) the secretion of cytokines and chemokines from activated BM-derived FSP-1^+^ cells is suppressed by KO of RBP-Jκ.

Organ fibrosis is the consequence of the healing response following inflammation. The increased infiltration of macrophages and production of cytokines or chemokines are prominent features in biopsy specimens of patients with CKD that are correlated with interstitial fibrosis [23]. Recent work exploring the nature of both circulating monocytes and tissue macrophages has highlighted their multifaceted phenotype and roles in renal fibrosis in vivo [24]. It has been proposed that proinflammatory macrophages directly promote renal fibrosis [25]. Increased expression of cytokines is associated with fibrosis in human diseases, including IgA nephropathy, lupus nephritis, and diabetic nephropathy [26,27,28].

Increased accumulation of FSP-1^+^ cells is associated with tissue fibrosis and inflammation in different organs [29,30,31]. FSP-1 has been used as a marker of fibroblasts in models of cardiac and liver fibrosis and as a marker of macrophages in mouse kidneys undergoing remodeling [10,32]. We found that FSP-1-positive cells isolated from injured mouse kidneys are of hematopoietic origin and belong to the myeloid-monocytic lineage. Regardless, FSP-1-positive cells express CD45 and macrophage marker, F4/80. We used FSP-1-GFP reporter mice to label FSP-1-positive cells and found that only a few GFP^+^ cells express the mesenchymal marker α-SMA. In addition, using genetic lineage tracing and BM transplantation experiments, we showed that FSP-1 does not colocalize with α-SMA, excluding the possibility that FSP-1 might be a specific marker of uncommitted myofibroblasts. Furthermore, most F4/80^+^ macrophages/monocytes express GFP in FSP-1-GFP reporter mice. These findings are consistent with previous studies showing that FSP-1 is not a marker of myofibroblasts in injured kidneys induced by thiazides and glomerulonephritis [9,33]. Thus, FSP-1 is not a marker of myofibroblast populations in obstructed kidneys.

FSP-1 positive cells express macrophage markers and secrete cytokines in fibrogenic kidneys. Macrophages are classified into two polarization states: M1 (classically activated) and M2 (alternatively activated) types [34]. M1 macrophage accumulation is associated with worse kidney parameters in a proinflammatory model of renal disease [35]. In RBP-Jκ-deficient macrophages, p65 nuclear translocation and Erk1/2 phosphorylation decreased while the level of IκB increased, suggesting that Notch-RBP-Jκ might regulate these inflammatory pathways in macrophages [36,37]. Disruption of RBP-Jκ in myeloid cells inhibits ECM deposition and myofibroblast activation during renal injury [38]. Interestingly, we found that Notch activity is required for the differentiation and activation of FSP-1^+^ cells into M1 macrophages but not M2 macrophages. LPS-stimulated macrophages upregulate MCP-1, IL-1β, iNOS, and IL-6; this induction could be blocked by a γ-secretase inhibitor or an RBP-Jκ knockdown, indicating that a Notch/RBP-Jκ signaling dependent pathway regulates the differentiation and activation of BM-derived FSP-1^+^ cells.

By using a conditional Cre transgenic mouse model (FSP-1^RBP-Jκ KO^), we found that the specific deletion of RBP-Jκ in FSP-1^+^ cells alleviates UUO-induced renal fibrosis. Moreover, inducible overexpression of DNMAML1 in FSP-1^+^ macrophages also reduced the inflammation in the kidneys. Therefore, these findings highlight that Notch signaling regulates macrophage polarization and cytokine secretion in an RBP-Jκ-dependent manner. In addition to reduced macrophage infiltration and MCP-1 expression, the production of inflammatory and fibrogenic cytokines IL-12β, IL-6, and IL-1β decreased upon RBP-Jκ specific deletion in FSP-1 cells in vivo and in FSP-1^+^ BM cells.

In conclusion, our study reveals that most FSP-1^+^ cells in obstructed kidneys are activated macrophages that are derived from BM. Notch signaling is required for the differentiation of BM-derived FSP-1^+^ into type 1 macrophages. Blocking Notch activation in FSP-1 cells inhibits the production of cytokines secreted from the FSP-1 cells and ameliorates renal inflammation and fibrosis.

## Data Availability

Not applicable.

## Supplementary Materials

The following are available online at https://www.mdpi.com/article/10.3390/cells12020214/s1, Figure S1: Scheme of breeding strategy to create RBP-Jκ^LoxP/LoxP^/STOP^LoxP/LoxP^-GFP/FSP-1-Cre^+/−^ mice. Figure S2: Scheme of breeding strategy to create DNMAML1/FSP-1-Cre transgenic mouse. Table S1: The primers used for RT-PCR.

## Abbreviations

UUO: unilateral ureteral obstruction; FSP-1, fibroblast specific protein 1; ECM, extracellular matrix protein.
