# Supplementary material for: Notch Blockade Specifically in Bone Marrow-Derived FSP-1-Positive Cells Ameliorates Renal Fibrosis
Source: Cells. 2023 Jan 4;12(2):214. doi: 10.3390/cells12020214 (PMC9856686; doi:10.3390/cells12020214)
Supplement: Supplementary file 1 [file cells-12-00214-s001.zip › cells-2082926-supplementary.pdf]

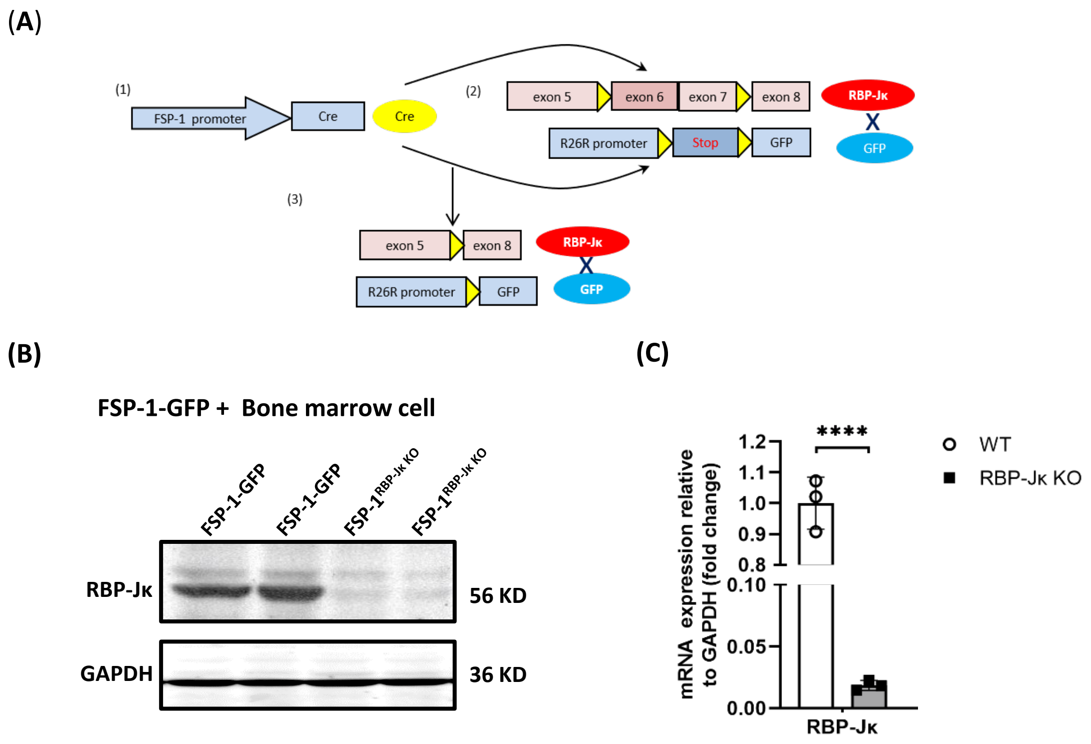

Supplemental Figure S1.Gene constructs present in RBP-Jκ $^{LoxP/LoxP}$/ GFP-STOP$^{LoxP/LoxP}$/FSP-1-Cre$^{+/-}$ mice.

(A) Triple transgenic mice: RBP-Jκ $^{LoxP/LoxP}$/GFP-STOP$^{LoxP/LoxP}$/FSP-1-Cre$^{+/-}$. (B) The GFP + BMCs were sorted by flow cytometry from FSP-1^{GFP} and FSP-1$^{RBP\text{-}J\kappa\ KO}$ mice, cell lysates were collected and the levels of indicated molecules were determined by western blots. (C) The expression of RBP-Jκ in WT and *KO* kidney were detected by qRT-PCR. Relative mRNA amount of RBP-Jκ in GFP^{+} BMCs from FSP-1^{GFP} and FSP-1$^{RBP\text{-}J\kappa\ KO}$ mice were analyzed .(**** , $P < 0.0001$ FSP-1^{GFP} vs FSP-1$^{RBP\text{-}J\kappa\ KO}$). Data are expressed as mean ± SEM, n = 3 mice.

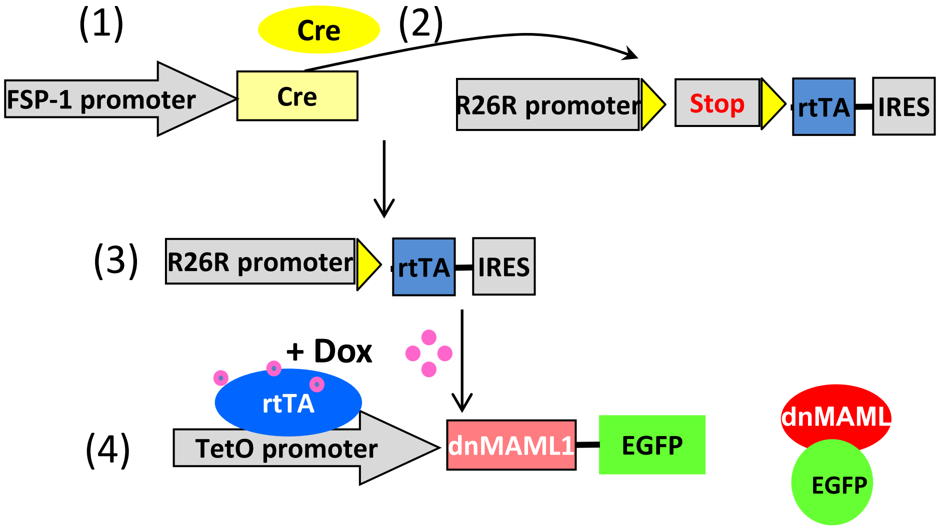

Supplemental Figure S2 .Gene constructs present in dnMAML1/ FSP-1/GFP triple transgenic mouse

Supplemental Table S1. The primers were designed for mouse

Notch 1	forward 5’ -TGTTCTTTGTGGGCTGTGG -3’<br>reverse 5’ -TAGGGTAACGGACAGGGATG -3’
Notch 2	forward 5’ - AAC TGGAGAGTCCAAGAAACG -3’<br>reverse 5’ - TGGTAGACCAAGTCTGTGATGAT-3’
Notch 3	forward 5’- CACCTTGGCCCCCTAAG -3’<br>reverse 5’ - TGG AATGCAGTGAAGTGAGG-3’
Notch 4	forward 5’ - CAAGCTCCCGTAGTCCTACTTC -3’<br>reverse 5’ - GGCAGGTGCCCCCATT -3’
DLL4	forward 5’ - AGGTGCCACTTCGGTTACAC-3’<br>reverse 5’- GGGAGAGCAAATGGCTGATA -3’
Jagged 1	forward 5’- TCTCTGACCCCTGCCATAAC-3’<br>reverse 5’ -TTGAATCCATTCACCAGATCC-3’
RBP-Jκ	forward 5’ -TGCCAAGGTGGCTCAGAAATCCTA-3’<br>reverse 5’ - ATTGGAGGGCTTGGTCTTGCATTG-3’
Hey 1	forward 5’-CATGAAGAGAGCTCACCCAGA-3’<br>reverse 5’-CGCCGAACTCAAGTTTCC-3’
Hey 2	forward 5’-GAGGAAACGACCTCCGAAA-3’<br>reverse 5’-GACCTCATCACTGAGCTTGTAGC -3’
Hes1	forward 5’-ACACCGGACAAACCAAAGAC -3’<br>reverse 5’ -CGCCTCTTCTCCATGATAGG-3’
Fibronectin	forward 5′-TTCCAGAAGGTGATGAG-3′<br>reverse 5′-TCATGGCAATGCAGGACAGGAAGA-3′
Collagen I	forward 5′-TTCCAGAAGGTGATGAG-3′<br>reverse 5′-TCATGGCAATGCAGGACAGGAAGA-3′
α-SMA	forward 5′-CTGACAGAGGCACCACTGAA-3′<br>reverse 5′-GAAATAGCCAAGCTCAG-3′
GAPDH	forward 5′-AGTGGGAGTTGCTGTTGAAATC-3′<br>reverse 5′-TGCTGAGTATGTCGTGGAGTCTA-3′
